# Cerebral Air Embolism as Possible Cause of Stroke During Therapeutic Endobronchial Application of Argon Plasma Coagulation

**DOI:** 10.7759/cureus.1255

**Published:** 2017-05-17

**Authors:** Venkatkiran Kanchustambham, Manasa Reddy, Swetha Saladi, Setu Patolia

**Affiliations:** 1 Pulmonary and Critical Care Medicine, Saint Louis University School of Medicine; 2 General Internal Medicine, Saint Louis University School of Medicine

**Keywords:** air embolism, cerebral air embolism, venous air embolism, arterial air embolism, paradoxical air embolism, broncho-vascular fistula

## Abstract

A 68-year-old male was admitted for evaluation of an endobronchial mass obstructing the right middle lobe (RML) and right lower lobe (RLL) of the lung. Flexible bronchoscopy revealed a notable endobronchial lesion in the bronchus intermedius that completely obstructed the RML and the RLL. Argon plasma coagulation (APC) at 30 watts and gas flow at 0.8 liters/minute to 1 liter/minute were applied to the tumor. In the recovery room, the patient was discovered to have a left-sided facial droop and left-sided weakness. The initial computed tomography (CT) scan of the brain and an angiogram of the head and neck were normal, but a repeat CT scan of the head several hours later was remarkable for an area of hypoattenuation in the right frontoparietal lobe concerning for infarct. A magnetic resonance imaging (MRI) brain scan confirmed acute to sub-acute cortical infarcts. Given the direct temporal relation between the onset of neurologic symptoms and the usage of APC with bronchoscopy, a cerebral air embolism (CAE) was thought to be the cause of the patient’s acute stroke.

## Introduction

Vascular air embolism (VAE) is a rare but potentially fatal complication of bronchoscopy and is most frequently reported with therapeutic bronchoscopy, argon plasma coagulation (APC), or neodymium-doped yttrium aluminum garnet (Nd-YAG) laser [1-2]. Despite VAE being rare (as a result of its high chance of mortality and morbidity), there needs to be high clinical suspicion to warrant immediate recognition and treatment. VAE can occur in either the venous or arterial system depending on the point of air entry into the systemic circulation. A venous air embolism occurs when gas enters a venous structure and progresses through the right heart to the pulmonary vessels. An arterial embolism results when air enters into the pulmonary veins or directly into the arteries of the systemic circulation resulting in embolization to the cerebral or coronary circulation [3].

Bronchoscopic APC resulting in VAE leading to cardiovascular collapse and cerebral air embolism (CAE) has been reported in various case reports previously. Reddy, et al. reported three cases of arterial air embolism leading to intracardiac gas embolism after APC. Goldman, et al. reported a case of cardiac arrest from arterial air embolism leading to left ventricular gas embolism after bronchoscopic APC. Yasmeen, et al. first reported a case of arterial air embolism leading to CAE from APC [4-6]. In this paper, we report the second case of CAE from bronchoscopic APC and discuss the several potential mechanisms responsible for causing CAE during APC.

## Case presentation

The patient was a 68-year-old male with squamous cell cancer with metastasis to the pleura that had been diagnosed and treated with two cycles of palliative chemotherapy four weeks before admission. The patient’s chronic medical conditions included chronic obstructive lung disease, hypertension, and continued tobacco use. The patient presented with shortness of breath on exertion and associated cough. He was found to have an endobronchial mass that was obstructing the right middle lobe (RML) and the right lower lobe (RLL) of the lung with resulting collapse of the RLL.

**Figure 1:**
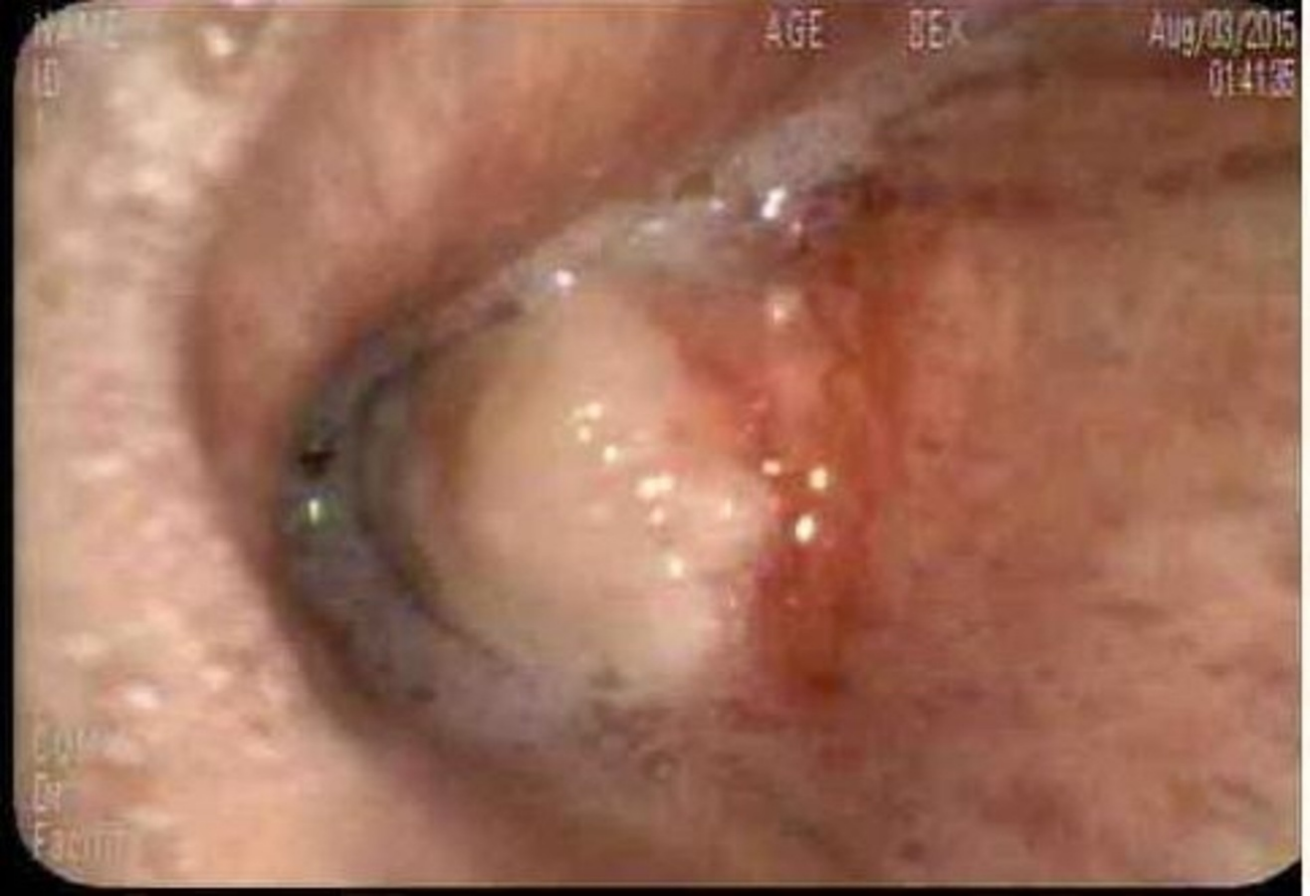
Large endobronchial lesion in the bronchus intermedius completely obstructing the right middle lobe and the right lower lobe

Flexible bronchoscopy was performed on the patient in the semi-recumbent position under moderate sedation during which the patient received 200 mcg of fentanyl and 7 mg of midazolam. The procedure revealed a large endobronchial lesion in the bronchus intermedius that completely obstructed the RML and the RLL (Figure 1). APC at 30 watts and gas flow at 0.8 liters/minute were applied to the tumor, followed by blunt dissection of devitalized tissues with cupped and rat tooth forceps. The blunt dissection resulted in moderate bleeding that was controlled with cauterization. The patient tolerated the four-hour procedure well and was then transferred to the recovery room.

On arrival at the recovery room, the patient was found to be drowsy and lethargic. These symptoms were initially thought to have been caused by the sedation administered during the procedure. A few hours later on repeat neurologic exam, the patient was more alert but was found to have a left-sided facial droop and left hemiplegia. As a result, the patient had a computed tomography (CT) scan of the brain and an angiogram of the head and neck. These studies did not show any findings consistent with acute stroke, hemorrhage or arterial occlusion. Despite this, there was a concern for a right middle cerebral artery (MCA) stroke given the clinical presentation.

**Figure 2:**
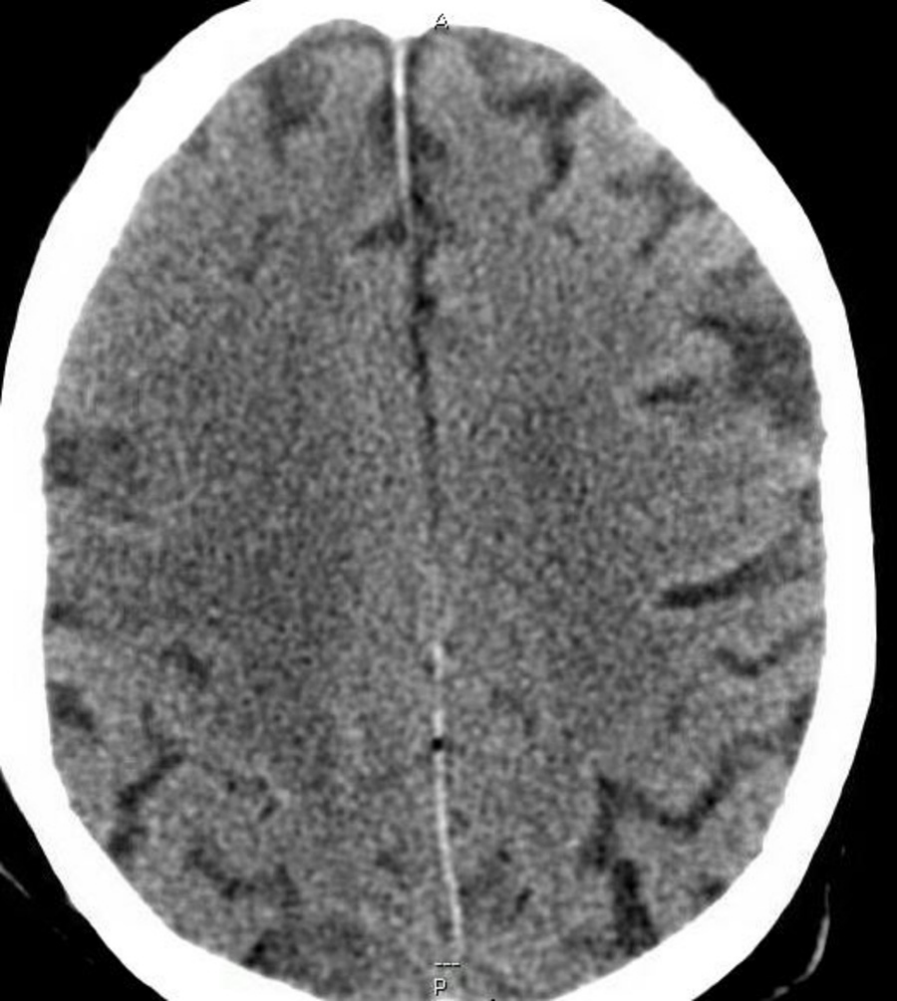
Area of hypoattenuation in the right frontoparietal lobe

The patient was admitted to the neurological intensive care unit (NICU) and was not given intravenous thrombolytics for the suspected stroke as he had sustained moderate bleeding with the bronchoscopy. Later that night, the patient had generalized tonic-clonic seizures that were aborted with benzodiazepines and levetiracetam. The patient then underwent repeat CT and magnetic resonance imaging (MRI) scan of the brain with and without contrast. The CT scan showed an area of hypoattenuation in the right frontoparietal lobe with a loss of gray-white matter differentiation concerning for an infarction in the right MCA territory without evidence of hemorrhagic conversion (Figure 2). 

**Figure 3:**
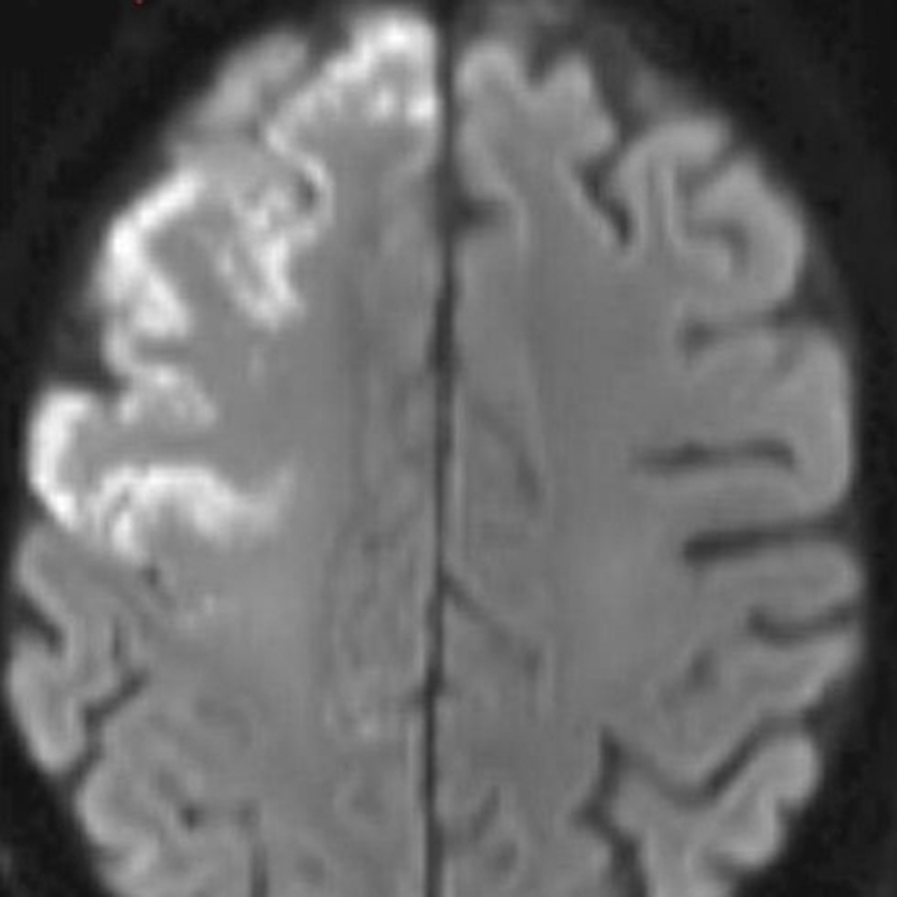
Acute to subacute cortical infarcts involving the right frontal lobe

The MRI brain scan showed acute to sub-acute cortical infarcts that involved the right frontal lobe in the right MCA territory without mass effect or evidence of hemorrhagic conversion (Figure 3.) Also, a transthoracic echocardiogram was done that showed no intracardiac shunt or thrombus.

The patient was placed on 100% oxygen and transferred to an outside facility for hyperbaric oxygen therapy. The patient’s mental status subsequently improved back to baseline but with a residual left-sided weakness. The patient was later discharged to a long-term rehabilitation facility. 

## Discussion

Bronchoscopic APC in this patient resulted in an altered level of consciousness and left sided weakness. This clinical deterioration, associated with generalized seizures, was most likely due to the development of CAE causing multiple end-arterial acute infarcts. In our patient, even though imaging studies were negative for cerebral air, we hypothesized that CAE was the likely cause of the acute stroke given the direct temporal relation between the onset of the symptoms and the use of APC. A thromboembolic cause of stroke cannot be excluded given the various vascular risk factors. 

The possible mechanisms responsible for the development of CAE during the APC include paradoxical embolization, formation of broncho vascular fistula, and occlusion of the bronchus by the bronchoscope.

Paradoxical embolization occurs during the APC when the gas flows from the venous circulation into the systemic circulation via the intracardiac shunts or by causing the filtering capacity of the pulmonary arterioles and capillaries to be overwhelmed by the rapid influx of large volumes of air [4-7]. In a dog model, it was noted that when 30 mL of air was injected rapidly, it produced embolization into arterial circulation resulting in cardiovascular compromise even in the absence of a patent foramen ovale [8]. This model applies to the case at hand as it is speculated that the rapidly infused argon gas or the gasses formed from superheated blood could have provoked a CAE in the absence of intracardiac shunts.

Bronchovascular fistulae are abnormal communications created between the pulmonary vein and airways due to heat coagulation and mechanical destruction of the tumor and adjacent tissue. High pressures formed in the airways due to positive pressure ventilation can result in air being forced from the airways into the pulmonary circulation via broncho vascular fistulae [4-7]. In our patient, APC ablation of the large tumor accompanied by mechanical debridement of the airway might have produced broncho vascular fistulae via which gas could have entered the arterial circulation.

Also, when the tip of a bronchoscope is advanced through a previously obstructed bronchus, it results in a post-obstructive high pressure that can force air directly from the airways into the pulmonary arteries through a broncho vascular fistula [4-7]. The patency of the airways accomplished after ablation was assessed in our patient by advancing the bronchoscope into the airways. This may have resulted in a transient occlusion of the airways that forced air across a fistula that could have formed during the mechanical debridement.

Air embolism can manifest with symptoms and presentations such as cardiac arrest, stroke, chest pain, paresthesias, convulsions, paralysis, seizures, visual disturbances, and headache. Regarding CAE, the CT or MRI scan findings may support or confirm the diagnosis but it is critical to note that normal imaging studies cannot be used to rule out CAE [1-2]. In a dog model, the CT scan detected only 20% of CAE when 0.25 mL of air was injected, and 2 mL of injected air was required before the CT was 100% sensitive for CAE [9]. Moreover, in various studies done in deep-sea divers who presented with neurologic and pulmonary symptoms of VAE, no CT evidence of cerebral air was demonstrated [10]. As a result, clinical evaluation is still preferred for the assessment of CAE. In our patient, even though imaging studies were negative for cerebral air, we hypothesized that CAE was the likely cause of the acute stroke given the direct temporal relation between the onset of the symptoms and the use of APC.

In the majority of cases, the diagnosis of CAE is speculated when there is a protracted recovery from general anesthesia or a temporary phase of impaired consciousness indicating the possible occurrence of CAE. The presence of residual anesthetic or muscle relaxant can mimic CAE which makes it difficult to establish the diagnosis. In our patient, there was a delay in diagnosis for approximately four hours before the stroke was suspected as the patient was not awake enough to perform a neurologic exam.

Patients suspected of having air embolism should be transferred to the intensive care unit (ICU) for careful monitoring and management. Hemodynamic support is the mainstay in the acute treatment of air embolism. Volume expansion with intravenous fluids is not only essential for resuscitation but also to avoid a wide pressure gradient that can increase the risk of air embolism.The patients should be placed on 100% oxygen to reduce the air bubble size and aid in the reabsorption of nitrogen gas from the bubble into the blood. Hyperbaric oxygen therapy involves the patient breathing 100% oxygen at a higher pressure than atmospheric sea level and is indicated in the presence of neurological deficits.

## Conclusions

Physicians should be aware of the occurrence of CAE as a possible complication of bronchoscopic APC. These patients can manifest with delayed recovery times from general anesthesia or sedation, new neurologic findings, or new cardiopulmonary instability. Some measures that can be used to reduce rates of complication are to set the flow to the lowest rate possible when using Nd-YAG or APC, avoid advancing the bronchoscope such that it occludes the bronchus, use the non-contact technique when using APC and avoid pushing the bronchoscope tip into or through the tumor. 
